# Development of an UPLC-MS/MS Method for the Analysis of Mycotoxins in Rumen Fluid with and without Maize Silage Emphasizes the Importance of Using Matrix-Matched Calibration

**DOI:** 10.3390/toxins11090519

**Published:** 2019-09-07

**Authors:** Sandra Debevere, Siegrid De Baere, Geert Haesaert, Michael Rychlik, Veerle Fievez, Siska Croubels

**Affiliations:** 1 Department of Pharmacology, Toxicology and Biochemistry, Faculty of Veterinary Medicine, Ghent University, Salisburylaan 133, 9820 Merelbeke, Belgium; 2 Department of Animal Sciences and Aquatic Ecology, Faculty of Bioscience Engineering, Ghent University, Coupure links 653, 9000 Ghent, Belgium; 3 Department of Plants and Crops, Faculty of Bioscience Engineering, Ghent University, Coupure Links 653, 9000 Ghent, Belgium; 4 Chair of Analytical Food Chemistry, Technische Universität München, Maximus-von-Imhof-Forum 2, 85354 Freising, Germany

**Keywords:** mycotoxins, UPLC-MS/MS, rumen fluid, maize silage, matrix-matched

## Abstract

Ruminants are less susceptible to the effects of mycotoxins than monogastric animals as their rumen microbiota are claimed to degrade and/or deactivate at least some of these toxic compounds. However, the mycotoxin degradation is not well-known yet. For this, a sensitive, specific, and accurate analytical method is needed to determine mycotoxins in the rumen fluid. This study aims to develop and thoroughly validate an ultra-performance liquid chromatography tandem mass spectrometry method for the quantitative determination in the rumen fluid of some of the most relevant mycotoxins found in maize silage in Western Europe: deoxynivalenol (DON), nivalenol (NIV), zearalenone (ZEN), mycophenolic acid (MPA), roquefortine C (ROQ-C) and enniatin B (ENN B), as well as their metabolites deepoxy-deoxynivalenol (DOM-1), α-zearalenol (α-ZEL), β-zearalenol (β-ZEL), zearalanone (ZAN), α-zearalanol (α-ZAL) and β-zearalanol (β-ZAL). As feed is often present in the rumen fluid samples, the potential interaction of feed particles with the mycotoxin extraction and analysis was investigated. Extraction recovery and matrix effects were determined in the rumen fluid with and without maize silage. Differences in those parameters between rumen fluid alone and rumen fluid with maize silage highlight the importance of using matrix-matched calibration curves for the quantification of mycotoxins in rumen fluid samples. A cross-validation of the method with rumen fluid and maize silage demonstrates that this analytical method can be applied in research on rumen fluid samples to investigate the degradation of the reported mycotoxins by rumen microbiota if matrix-matched calibration is performed.

## 1. Introduction

Mycotoxins are secondary fungal metabolites that are harmful to humans and/or animals. Monogastric animals, in particular pigs, are more susceptible to toxic effects of mycotoxins than ruminants as the symbiosis of ruminants with rumen microbiota allows (partial) mycotoxin degradation [1,2]. Despite this potential degradation, mycotoxin-associated subclinical health problems have been reported in high-productive dairy cows, reflected by vague and non-specific symptoms and periodic decrease in milk production [1]. The increased milk yield during the past decades is not only associated with a higher incidence of metabolic disorders and hence a lower detoxifying capacity of mycotoxins in the rumen [3], but also with a higher rumen passage rate and a higher proportion of highly fermentable maize silage in the ration of cows. Unfortunately, this maize silage is more vulnerable to contamination with multiple mycotoxins than e.g., grassland products [4,5,6].

In Belgium, which has a temperate climate, some of the most important mycotoxins found in maize silage are deoxynivalenol (DON), nivalenol (NIV), zearalenone (ZEN), mycophenolic acid (MPA), roquefortine C (ROQ-C), and enniatins, especially enniatin B (ENN B) [4,7,8,9] (Tables S1 and S2). However to determine their rumen degradation, a sensitive, specific, accurate and high-throughput multi-method allowing analysis of these mycotoxins and their metabolites in rumen fluid is essential.

Nowadays, ultra-performance liquid chromatography (UPLC) combined with tandem mass spectrometry (MS/MS) offers a powerful tool in the analysis of mycotoxins, as this method offers a high specificity and sensitivity and can detect low levels of mycotoxins in complex matrices. Hence, ultra-performance liquid chromatography tandem mass spectrometry (UPLC-MS/MS) has been used for the detection of several types of mycotoxins in different matrices such as food and feed [10,11,12], blood [13] and urine [14,15].

The analysis of mycotoxins in rumen fluid, however, is highly challenging because of the complex nature of the matrix, including partially degraded feed particles and its microbiota. First, an elaborate sample preparation before chromatographic analysis is crucial in the development of such a detection method to eliminate as much as possible the unwanted compounds that can interfere with the detection of the analytes. In addition, in case of multi-mycotoxin methods, it is difficult to develop an analytical method for the simultaneous determination of several mycotoxins, since they have different physicochemical properties. Hence, compromises have to be made in optimizing the sample preparation and MS parameters. Second, it has been described that bacteria and yeast cell walls, also present in rumen fluid, are able to bind mycotoxins such as DON, ZEN, aflatoxins and fumonisins leading to an inferior detection of free mycotoxins in the rumen fluid [16,17,18,19,20,21,22,23,24].

To the authors’ knowledge, very few methods have been described to determine simultaneously several mycotoxins in rumen fluid and none of them included all mycotoxins that are often found in maize silage (DON, NIV, ZEN, MPA, ROQ-C, and ENN B). Kiessling et al. (1984) analyzed six different mycotoxins (aflatoxin B1, ochratoxin A, ZEN, T-2 toxin, diacetoxyscirpenol, and DON) in the rumen fluid [25]. However, to determine those mycotoxins from different classes, four different extraction procedures and detection methods were performed, which are time consuming and expensive. Gallo et al. (2015) determined simultaneously three *Penicillium roqueforti* mycotoxins (MPA, ROQ-C and *Penicillium roqueforti* toxin (PR toxin)) in the rumen fluid by means of HPLC-MS/MS [26]. Again, the extraction procedure was very time consuming as the purpose of the study was to distinguish between mycotoxins present in the supernatant of the rumen fluid and those present in the pellet of the rumen fluid, so two separate extractions were performed.

Therefore, the goal of this study is first to develop a high-throughput method for rumen fluid to extract the most prevalent and toxicological important mycotoxins found in maize silage (DON, NIV, ZEN, MPA, ROQ-C, and ENN B) and their metabolites (deepoxy-deoxynivalenol (DOM-1), α-zearalenol (α-ZEL), β-zearalenol (β-ZEL), zearalanone (ZAN), α-zearalanol (α-ZAL), and β-zearalanol (β-ZAL)) as these can be formed in the rumen [25,27,28,29,30,31]. Further, a highly specific and sensitive UPLC-MS/MS method to quantify these components in the rumen fluid was developed and validated. As rumen fluid is a complex matrix and mycotoxins can bind to feed or other particles present in rumen fluid, the impact of the matrix on extraction recovery and matrix effects was evaluated. As the importance of using matrix-matched calibration curves was evident, a cross-validation of the method with rumen fluid and maize silage as a matrix was performed to prove the usability of the method in the research on rumen fluid samples if matrix-matched calibration is used.

## 2. Results and Discussion

Several criteria were taken into account when developing the UPLC-MS/MS method. The sample preparation procedure had to be fast and straightforward to ensure an acceptable daily throughput (sample no. >140). Along with this, a run-time of ≤10 min per ionization mode (electrospray (ESI) positive (+) or negative (−)) was aimed for. However, simplicity and speed of analysis should not be reached at the expense of reliability, specificity, and sensitivity. Finally, a thorough method validation was performed prior to routine application. Special attention was paid to the potential interaction of feed particles, in this study on maize silage, with the extraction and analysis of mycotoxins, which is important to setup an appropriate calibration method.

### 2.1. Mycotoxin Selection

Mycotoxin concentrations in the rumen can be very low, certainly when kinetic degradation studies are performed as concentrations might gradually decrease. Hence, the developed UPLC-MS/MS method needs to be very sensitive to detect and quantify mycotoxins and their metabolites. As the sensitivity which can be reached by a multi-method also partially depends on the number of mycotoxins included, a selection of the most important mycotoxins found in maize silage in Western Europe was made. Sensitivity results are reported in Section 2.4.3. Limit of Quantification (LOQ) and Limit of Detection (LOD).

### 2.2. UPLC-MS/MS Method

Mass spectrometric parameters of both the precursor and product ions were tuned in their most sensitive ESI mode (+/−). For each compound, two specific product ions were selected from the mass spectrum after fragmentation (Table 1). The product ion with the highest intensity was selected for quantification, whereas the second most intense product ion was used for qualification.

**Figure 1:**
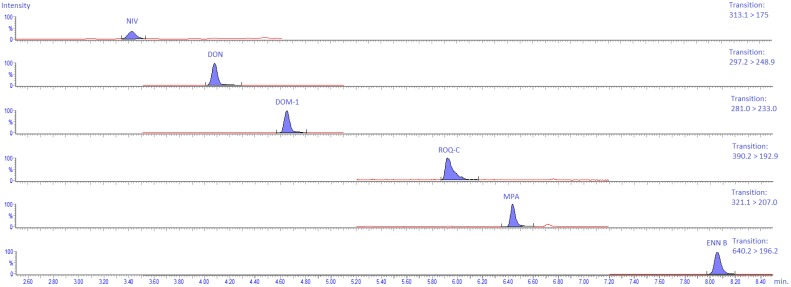
UPLC-MS/MS chromatograms of the analysis of nivalenol (NIV), deoxynivalenol (DON), deepoxy-deoxynivalenol (DOM-1), roquefortine C (ROQ-C), mycophenolic acid (MPA), and enniatin B (ENN B) in a rumen fluid–buffer mixture spiked at a level of 600 ng/mL (NIV), 120 ng/mL (DON), 122 ng/mL (DOM-1), 20 ng/mL (ROQ-C), 60 ng/mL (MPA), and 10 ng/mL (ENN B). These mycotoxins are detected in ESI+ mode. For each analyte, only the transition of the precursor ion to the product ion with the highest intensity (quantifier) is shown.

**Figure 2:**
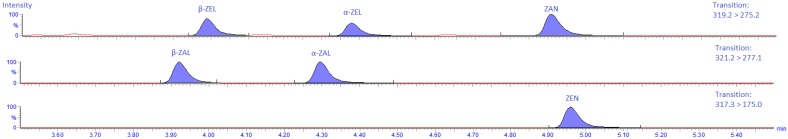
UPLC-MS/MS chromatograms of the analysis of zearalenone (ZEN) and its major metabolites in a rumen fluid–buffer mixture spiked at a level of 30 ng/mL of ZEN, zearalanone (ZAN), α-zearalenol (α-ZEL), β-zearalenol (β-ZEL), α-zearalanol (α-ZAL), and β-zearalanol (β-ZAL). These mycotoxins are detected in ESI− mode. For each analyte, only the transition of the precursor ion to the product ion with the highest intensity (quantifier) is shown.

Different columns, mobile phases, LC gradient programs and flow rates were evaluated in terms of resolution, peak intensity and shape, and signal-to-noise (S/N) ratio (data not shown). Four different reversed-phase columns were tested (A/ Acquity UPLC BEH C18, Waters; B/ Hypersil gold, ThermoScientific; C/ Acquity UPLC HSS T3 column, Waters; D/ Zorbax Eclipse Plus, Agilent). The best results were obtained with the Acquity UPLC^®^ HSS T3 column (100 mm × 2.1 mm I.D., 1.8 µm particle size). Most mobile phases described in literature consist of a combination of water and methanol (MeOH) or acetonitrile (ACN) to which some additives can be added to improve chromatography and detection [32]. A better sensitivity was observed for DON when MeOH was used. For an optimal chromatographic separation of ZEN and its metabolites, ACN was needed [33]. Several concentrations of ammonium acetate and/or acetic acid or formic acid were tested. The mobile phases with their LC gradient program that showed the best results were finally chosen for the method are shown in Table 2. Because of the diverse physicochemical characteristics of the mycotoxins, it was not possible to analyze all mycotoxins in the same run. Hence, to detect mycotoxins in ESI− and ESI+ mode, the samples had to be analyzed by two subsequent analytical runs, within a total run-time of 17 min. Chromatographic separation of ZEN and its metabolites was performed in ESI− mode within a time frame of 7 min using a gradient elution of H_2_O and ACN as mobile phases (MP); DON, DOM-1, NIV, ENN B, MPA, and ROQ-C were analyzed in ESI+ mode within a run-time of 10 min, using a gradient elution of 0.01% acetic acid (*v*/*v*) in H_2_O and 0.01% acetic acid (*v*/*v*) in MeOH as MP. Figure 1 and Figure 2 show the chromatograms of the mycotoxins present in a rumen fluid–buffer mixture after extraction, determined in ESI+ and ESI− mode, respectively. In comparison with the run-time of Gallo et al. (2015) to detect PR toxin, MPA, and ROQ-C in the rumen fluid by means of HPLC-MS/MS, the run-time for the positive ionization mode is 2 min shorter while 12 mycotoxins with various physicochemical properties were determined [26].

### 2.3. Sample Preparation and Extraction

The use of a simple and practical sample preparation procedure is desirable in order to reduce the time and cost of analysis. Theoretically, clean-up of samples can be kept to a minimum when analytical methods as sensitive and specific as UPLC-MS/MS are applied. However, the “dilute-and-shoot” principle was not considered in this study because of the complexity of the rumen fluid and the higher chance of clogging and pollution of the MS instrument. On the other hand, the more sophisticated extraction procedure with solid phase extraction (SPE) was not recommended as this seemed to be rather expensive and time-consuming, especially when large numbers of samples have to be analyzed. A liquid–liquid extraction (LLE) was chosen as an alternative and was a good compromise because of the simplicity of extraction, high throughput sample preparation procedure, and satisfactory sample clean-up. In the context of “green analytical chemistry” (GAC), ethyl acetate (EtAc) was chosen as the extraction solvent as this is a more environmentally friendly and cheaper alternative for ACN, which is often used in mycotoxin extraction protocols [34]. In addition, the simultaneous extraction of six mycotoxins and six mycotoxin metabolites minimizes the solvent consumption (250 µL PBS and 1.5 mL EtAc per sample) and chemical waste, and makes the chemical analysis more environmentally friendly [35]. In order to increase the extraction recovery, the volume of the organic extraction solvent was three times the volume of the aqueous phase (rumen fluid + buffer) as the distribution coefficient of the analytes between the organic and aqueous phase is dependent on their molar concentrations in the different phases. The combination of water and ACN as redissolution solvent at a 85/15 (*v*/*v*) ratio was crucial to obtain a good peak shape for all mycotoxins with their various physicochemical properties. Prior to UPLC-MS/MS analysis, the sample was passed through a filter to remove the remaining solid particles. For this step, several filters were tested. The Millex^®^-GV filter (0.22 µm, hydrophilic PVDF membrane, 13 mm) retained ROQ-C and ENN B and the Millex^®^-GN filter (0.20 µm, hydrophilic nylon membrane, 13 mm) retained MPA, ENN B, and ZEN and its metabolites. Only the Millex^®^-LG filter (0.20 µm, hydrophilic PTFE membrane, 4 mm) did not show any adsorption of mycotoxins.

### 2.4. Method Validation with Rumen Fluid–Buffer Mixture as Matrix

For this full method validation, a rumen fluid–buffer mixture was used as the matrix.

#### 2.4.1. Linearity

Linear calibration curves were obtained for each component over the concentration ranges tested (see Table 3).

The correlation coefficients (r) and goodness-of-fit coefficients (gof) met the acceptance criteria of ≥0.99 and ≤20%, respectively (Table 3).

The method of internal standardization was used to compensate for the analyte losses during sample preparation and for matrix effects during UPLC-MS/MS analysis. For DON, ENN B, MPA, ROQ-C, and ZEN, the corresponding isotopically labeled internal standard was used, respectively ^13^C_15_-DON, ^15^N_3_-ENN B, ^13^C_17_-MPA, ^13^C_22_-ROQ-C, and ^13^C_18_-ZEN. Although the use of an IS for every single mycotoxin included in a multi-mycotoxin method is preferable, this is not feasible in practice because of financial limitations or lack of commercial availability. In this case, structurally related standards were also used, e.g., ^13^C_15_–DON was chosen as IS not only for the analysis of DON, but also DOM-1 and NIV, and ^13^C_18_-ZEN was chosen not only for the analysis of ZEN but also for its metabolites, as they have very similar physicochemical properties [10,33]. This is justified as it has been demonstrated during the method validation experiments that a reliable determination of these compounds was not impaired.

#### 2.4.2. Accuracy and Precision

The within-run and between-run accuracy and precision were tested at three different concentration levels (limit of quantification (LOQ, see Section 2.4.3.), medium and high, see Table 3). The acceptability ranges for accuracy and within-run precision were met for all compounds at the specified levels according to VICH GL49 [36]. The between-run precision fell within the maximal allowed relative standard deviation (RSD_max_ = 2^(1–0.5logConc)^) [36]. An overview of the results is given in Table 4.

#### 2.4.3. Limit of Quantification (LOQ) and Limit of Detection (LOD)

The LOQ was set as the lowest concentration of the calibration curve that could be determined with an accuracy and precision within the acceptability ranges at the specified level. The LOQ ranged between 0.1 and 1.56 ng/mL, except for NIV that had an LOQ of 36 ng/mL (Table 3). The higher LOQ of NIV could be attributed to compromises that had to be made for a combined analysis of all analytes. Although NIV shows a better chromatographic response when determined in ESI- mode with mobile phases containing acetic acid [37], NIV is determined in ESI+ mode as adding acetic acid to the mobile phases led to a lower sensitivity for ZEN and its metabolites.

The LODs were theoretical and calculated based on the mean S/N ratios of the LOQ samples. Concentrations corresponding with a theoretical S/N ratio of 3 were set as the LOD and ranged from <0.01 to 0.17 ng/mL, except for NIV that had a LOD of 5.43 ng/mL.

In the study of Gallo et al. (2015) 15 mL of sample was used for mycotoxin determination with an LOD and LOQ of 4 and 10 ng/mL for both ROQ-C and MPA, which is much higher than the LOD of <0.01 ng/mL for ROQ-C and 0.28 ng/mL for MPA, and the LOQ of 0.1 ng/mL for ROQ-C and 0.6 ng/mL for MPA for the presented UPLC-MS/MS method that used only 250 µL of sample [26].

#### 2.4.4. Signal Suppression/Enhancement (SSE) and Extraction Recovery (R_E_)

The signal suppression/enhancement (SSE) was considered tolerable if the value ranged between 0.8 and 1.2 [38], with values outside this range indicating a strong matrix effect. SSE has been evaluated quantitatively and the results are shown in Table 5 (matrix A). As can be seen, there was a strong matrix effect for almost all analytes evaluated, which can be attributed to the complex matrix of rumen fluid. Extraction recoveries (R_E_) were highest for DON and DOM-1 (>40%), around 20% for ZEN and its metabolites, around 15% for ENN B and ROQ-C and low for NIV and MPA (<10%) (see Table 5). These variations in R_E_ can be attributed to the different physicochemical properties of the different mycotoxins which e.g., results in differences in adsorption to particles present in rumen fluid. Such low R_E_ is hypothesized to be partially linked to enhanced adsorption/binding to (feed) particles as it has been described that bacteria and yeast cell walls could be involved in binding aflatoxins, DON, ZEN, and fumonisins, [16,17,18,19,20,21,22,23,24]. In order to minimize the variations because of R_E_ and SSE, the use of matrix-matched calibration curves based on samples, which had been subjected to the whole analytical procedure and the use of isotope labeled ISs are thus mandatory to quantify the selected mycotoxins and their metabolites in rumen fluid with an acceptable accuracy and precision. Despite the strong matrix effects and low R_E_, very low LOD and LOQ values could be obtained because of the very sensitive UPLC-MS/MS analytical method.

#### 2.4.5. Specificity

**Figure 3:**
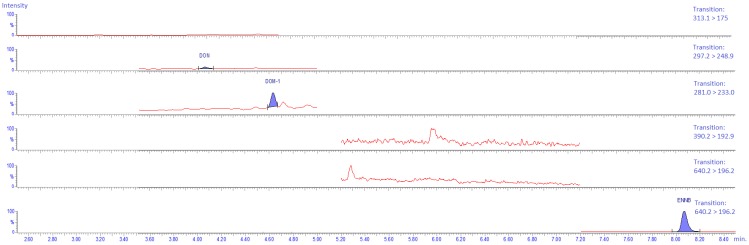
UPLC-MS/MS chromatograms of the analysis of nivalenol (NIV), deoxynivalenol (DON), deepoxy-deoxynivalenol (DOM-1), roquefortine C (ROQ-C), mycophenolic acid (MPA), and enniatin B (ENN B) in a rumen fluid–buffer mixture that was not spiked with mycotoxins. The rumen fluid was taken from a cow before morning feeding and showed traces of DON (0.45 ng/mL), DOM-1 (1.56 ng/mL), and ENN B (0.35 ng/mL). For each analyte, only the transition of the precursor ion to the product ion with the highest intensity (quantifier) is shown.

**Figure 4:**
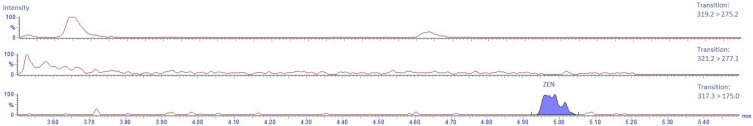
UPLC-MS/MS chromatograms of the analysis of zearalenone (ZEN) and its major metabolites zearalanone (ZAN), α-zearalenol (α-ZEL), β-zearalenol- (β-ZEL), α-zearalanol (α-ZAL), and β-zearalanol (β-ZAL) in a rumen fluid–buffer mixture that was not spiked with mycotoxins. The rumen fluid was taken from a cow before morning feeding and showed traces of ZEN (<LOQ). For each analyte, only the transition of the precursor ion to the product ion with the highest intensity (quantifier) is shown.

Prior to method validation, blank samples of the rumen fluid were analyzed to evaluate the presence/absence of mycotoxins. Small peaks could be detected at the elution zone of DON, DOM-1, ENN B, and ZEN, indicating that the rumen fluid already contained small amounts of these mycotoxins (DON: 0.45 ng/mL, DOM-1: 1.56 ng/mL, ENN B: 0.35 ng/mL, and ZEN: <LOQ) (Figure 3 and Figure 4). However, these “background” mycotoxin levels could be compensated for by the use of a matrix-matched calibration approach.

#### 2.4.6. Carry-Over

No carry-over of analytes from one sample to another was observed, except for ROQ-C (0.3% carry over). This can be attributed to the flow-through-needle (FTN) design of the Acquity H-Class system. In case of ROQ-C analysis, carry-over could be overcome by the injection of two solvent samples after each validation/study sample with a suspected high concentration of ROQ-C [39].

### 2.5. Evaluation of the Impact of Maize Silage on the Extraction Recovery (R_E_) and Signal Suppression/Enhancement (SSE)

As a substrate is always added to the rumen fluid to maintain fermentation during in vitro rumen incubations, and rumen fluid samples from in vivo studies also contain feed particles, feed particles can interact with the extraction and analysis of mycotoxins. Hence, the effect of feed particles, i.e., maize silage in this study, on R_E_ and SSE was investigated. The extraction recovery (R_E_) in rumen fluid–buffer mixture including 10 mg maize silage per mL (RF + MS) was compared with the corresponding R_E_ in rumen fluid–buffer mixture without maize silage (RF). The results are shown in Table 5. SSE is mainly negatively influenced by the addition of maize silage in the samples, which was indeed expected as maize silage makes the matrix more complex. Moreover, the R_E_ is influenced by the addition of maize silage, which can be attributed to a change in solubility of the mycotoxins by addition of maize silage and/or the differences in adsorption to feed particles. Therefore, it is of utmost importance to prepare matrix-matched calibration curves.

### 2.6. Cross-Validation of the Method with Rumen Fluid and Maize Silage as Matrix

To prove that the described UPLC-MS/MS method can also be used with rumen fluid samples that also include substrate, the method was subjected to a cross-validation whereby 10 mg of maize silage was added per mL of rumen fluid–buffer mixture. Linearity, within-run accuracy and within-run precision were determined in the same way as the full method validation with rumen fluid–buffer mixture alone. The results are shown in Table 6 and Table 7.

Similar to the full method validation with rumen fluid–buffer mixture, the correlation coefficients (r) and goodness-of-fit coefficients (gof) met the acceptance criteria of ≥0.99 and ≤20%, respectively (Table 6). In addition, the within-run accuracy and precision at three different concentrations (LOQ, medium, and high) met the acceptability ranges for all compounds (Table 7). The LOQ ranged from 0.2 to 7.99 ng/mL, except for NIV that had a LOQ of 36 ng/mL. The LOD ranged from 0.01 to 0.42 ng/mL, except for NIV that had a LOD of 9.28 ng/mL. The LOQ and LOD values are somewhat higher compared to the LOQ and LOD values of the rumen fluid–buffer mixture alone, which can be explained by the more complex matrix. In addition, for the cross-validation, following concentrations were found in the blank sample with maize silage: DON: 7.39 ng/mL, ENN B: 0.34 ng/mL, and ZEN: 0.46 ng/mL, which could be due to a higher contamination of the rumen fluid used for the cross-validation, but also from the maize silage that is already contaminated with small amounts of mycotoxins. The relative high concentration of DON in the blank sample explains also the higher LOQ of DON compared to the full validation method without maize silage. However, these values are still very low compared to other methods. The method of Gallo et al. (2015) had an LOD and LOQ of 4 and 10 ng/mL for both ROQ-C and MPA, which is much higher than the LOD of 0.01 ng/mL for ROQ-C and 0.42 ng/mL for MPA, and the LOQ of 0.2 ng/mL for ROQ-C and 1.2 ng/mL for MPA for the cross-validation with rumen fluid and maize silage [26].

These results demonstrate that this method can be used for the quantitative determination of mycotoxins and their relevant metabolites in the rumen fluid samples if matrix-matched calibration curves are used. For in vitro rumen simulation studies for example, this implies that a calibration curve has to be prepared with rumen fluid which contains the same substrate as used in the study.

## 3. Conclusions

This study describes the development and in-house validation of a sensitive and specific UPLC-MS/MS method for the quantification of the most relevant mycotoxins and their possible metabolites in the rumen fluid. The sample extraction procedure is simple and straightforward and consists of a liquid–liquid extraction using EtAc. Furthermore, the UPLC-MS/MS analysis of each sample is split in two analytical runs, i.e., in ESI+ and ESI− mode, with a run-time of 10 and 7 min, respectively, resulting in a total analysis time of 17 min/sample allowing a high throughput. The method is successfully validated for all analytes of interest according to international guidelines and literature. Furthermore, as feed particles are often present in rumen fluid samples, the interaction of maize silage on SSE and R_E_ was demonstrated which emphasizes the importance of using matrix-matched calibration curves to allow a correct quantification of analytes. A cross-validation of the method with rumen fluid and maize silage as matrix demonstrates that this analytical method can be applied in research to investigate the degradation of the reported mycotoxins by rumen microbiota in rumen fluid samples if matrix-matched calibration curves are used.

## 4. Materials and Methods

### 4.1. Mycotoxin Selection

Mycotoxin selection was based on their prevalence in Belgium and their toxicological impact. In 2016, maize samples from 91 maize fields across Flanders (Belgium) were analyzed on mycotoxin contamination before ensiling. The mycotoxins most often found in these samples were NIV, DON, ZEN, and ENN B and were found in respectively 98.9%, 92.3%, 64.8%, and 42.9% of all samples (Table S1). Of those samples, 2% and 1% exceeded the European Union reference values for DON and ZEN, respectively. Hence, NIV, DON, ZEN, ENN B and their metabolites DOM-1, ZAN, α-ZEL, β-ZEL, α-ZAL, and β-ZAL were selected as pre-harvest mycotoxins. Additionally, mycotoxin contamination data of 21 maize silages and 100 silage samples were used and mycotoxins that had a minimum prevalence of 90% in non-moldy and/or moldy parts were also included. In addition to DON (in all samples), ZEN (in 90% of the maize silage samples), and ENN B (in 91% of silage samples), ROQ-C (in all moldy hot spots) and MPA (95% in non-moldy parts) were highly prevalent (Table S2). Although citrinin was also often present in maize silage (95%), this mycotoxin was not included in the UPLC-MS/MS method as this mycotoxin needs acidified extraction solvents in contrast to the other mycotoxins [40].

### 4.2. Rumen Fluid, Maize Silage, Mycotoxins, Chemicals, and Reagents

Rumen fluid was collected from three fistulated dairy cows (Institute for Agricultural, Fisheries and Food Research, EC2014/241) prior to the morning feeding and immediately transferred to thermos flasks before making the rumen fluid–buffer mixture (see Section 4.3). Sampling was done for the full method validation and another sampling was performed for the cross-validation.

Maize silage was obtained from Agrivet (Melle, Belgium) in May 2012 and lyophilized. Mycotoxin concentrations were determined at the Centre of Excellence in Mycotoxicology and Public Health, Department of Bioanalysis at Ghent University, Belgium. Only small amounts of NIV (201 µg/kg), DON (593 µg/kg), ZEN (68 µg/kg), and ROQ-C (12 µg/kg) were detected. The concentration of ENN B was below the cut-off value of 80 µg/kg.

The analytical standards of DON, NIV, MPA, ROQ-C, and ZEN were purchased from Fermentek (Jerusalem, Israel). The standards of DOM-1, ENN B, ZAN, α-ZEL, β-ZEL, α-ZAL, and β-ZAL were purchased from Sigma-Aldrich (Overijse, Belgium). The internal standards (IS) ^13^C_17_-MPA, ^13^C_22_-ROQ-C, and ^13^C_18_-ZEN were purchased from Food Risk Management (Oostvoorne, The Netherlands). The IS ^13^C_15_-DON was purchased from Sigma-Aldrich. The IS ^15^N_3_-ENN B was kindly donated by the Chair of Analytical Food Chemistry at the Technical University of Munich (Freising, Germany) [41].

Methanol (MeOH), acetonitrile (ACN), ammonium acetate, and water (H_2_O) were purchased from Biosolve (Valkenswaard, The Netherlands) and were of UPLC/MS grade. Acetic acid (AA), hydrochloric acid (HCl) 37%, ethyl acetate (EtAc) were purchased from Merck Millipore (Overijse, Belgium) and were of analytical grade. Sodium chloride (NaCl), potassium dihydrogenphosphate (KH_2_PO_4_), and ammonium hydrogen carbonate (NH_4_HCO_3_) were purchased from VWR (Leuven, Belgium). The phosphate-buffered saline (PBS) powder packs were purchased from Thermo Fisher Scientific (Merelbeke, Belgium). Disodium hydrogen phosphate dodecahydrate (Na_2_HPO_4_·12H_2_O) and magnesium chloride hexahydrate (MgCl_2_·6H_2_O) were from Carl Roth (Vienna, Austria). Sodium hydrogen carbonate (NaHCO_3_) was purchased from Sigma-Aldrich. Carbon dioxide (CO_2_) 100% was purchased from Air Liquide (Aalter, Belgium).

### 4.3. Preparation of Standard Solutions and Rumen Fluid–Buffer Mixture

Stock solutions of 1 mg/mL in ACN were prepared for DON, NIV, MPA, ROQ-C, ZEN, and ENN B and of 100 μg/mL in ACN for ZAN, α-ZEL, β-ZEL, α-ZAL, and β-ZAL. DOM-1 was already in solution upon purchase (50 μg/mL in ACN). An IS-stock solution of 5 μg/mL in ACN was prepared for ^15^N_3_-ENN B. All other IS were already in solution upon purchase (^13^C_15_-DON: 25.3 μg/mL in ACN, ^13^C_17_-MPA: 25.4 μg/mL in ACN, ^13^C_22_-ROQ-C: 25 μg/mL in ACN, ^13^C_18_-ZEN: 25.4 μg/mL in ACN). The standard stock solutions were used to make a combined working solution of all standards in ACN without IS (3 µg/mL for DON and DOM-1, 15 µg/mL for NIV, 0.25 µg/mL for ENN B, 1.5 µg/mL for MPA, 0.5 µg/mL for ROQ-C, and 0.75 µg/mL for ZEN, ZAN, α-ZEL, β-ZEL, α-ZAL, and β-ZAL). Serial dilutions (10×, 100×, and 1000×) of the combined working solutions were prepared. A combined working solution of all IS was prepared with a final concentration of 100 ng/mL for ^13^C_15_-DON, ^13^C_17_-MPA, and ^13^C_18_-ZEN and 10 ng/mL for ^15^N_3_-ENN B and ^13^C_22_-ROQ-C. The standard and IS stock solutions were used for the preparation of matrix-matched calibrator and quality control (QC) samples. All stock and working solutions were stored at ≤−15 °C.

As in vitro rumen simulations are performed in a buffered rumen environment, a rumen fluid–buffer mixture was also used for the method development. The buffer contained the following compounds per liter: 3.58 g Na_2_HPO_4_·12H_2_O, 1.55 g KH_2_PO_4_, 0.124 g MgCl_2_·6H_2_O, 8.74 g NaHCO_3_, and 1.00 g NH_4_HCO_3_. The buffer was saturated with CO_2_ overnight and kept at 39 °C. The next day, fresh rumen fluid of three cows was mixed and sieved using a sieve with mesh width of 1 mm and added to the buffer at a rumen fluid/buffer ratio of 263.2 mL/1000 mL. The rumen fluid–buffer mixture was stored at ≤−15 °C until calibrator, validation and quality control (QC) samples were prepared.

### 4.4. Preparation of Calibrator, Validation and QC Samples

Matrix-matched calibrator samples were prepared by adding the appropriate combined working solution to 250 µL rumen fluid–buffer mixture with or without maize silage to obtain a calibration range of 0.24–180 ng/mL of DON and DOM-1, 1.2–900 ng/mL of NIV, 0.02–15 ng/mL of ENN B, 0.12–90 ng/mL of MPA, 0.04–30 ng/mL of ROQ-C and 0.06–45 ng/mL of ZEN, ZAN, α-ZEL, β-ZEL, α-ZAL and β-ZAL. QC samples were prepared at medium and high concentration levels (Table 3). For DON and ZEN, the high concentration levels were based on the recommended maximum values in maize by-products formulated by the Commission of the European Communities [42]. As no recommended maximum values are available for the other mycotoxins, the high concentration levels of those mycotoxins were based on the worst case contamination levels in maize silage found in Belgium/the Netherlands described in literature [4,7,8,9].

### 4.5. Mycotoxin Extraction

The following procedure was applied to extract DON, DOM-1, NIV, ENN B, MPA, ROQ-C, ZEN, ZAN, α-ZEL, β-ZEL, α-ZAL, and β-ZAL from the matrix-matched calibrator, validation or QC samples: a 250-µL aliquot of the rumen fluid–buffer mixture was taken and transferred to a polypropylene 15-mL extraction tube, followed by the addition of 25 µL of the IS-mix working solution, 250 µL PBS and 1.5 mL of EtAc. The samples were vortex mixed and extracted on an overhead shaker (15 min). After centrifugation (3724× *g*, 5 min, 4 °C), the upper organic phase was collected and evaporated to dryness under a gentle nitrogen stream at ~50 °C. The dry residue was redissolved in 200 µL of H_2_O/ACN (85/15, *v*/*v*), vortex mixed, filtered using a 0.20 µm Millex^®^-LG PTFE filter (Merck Millipore, Overijse, Belgium), and collected in an autosampler vial.

### 4.6. UPLC-MS/MS Analysis

The UPLC system consisted of an Acquity H-Class Quaternary Solvent Manager and Flow-Through-Needle Sample Manager with temperature-controlled tray and column oven from Waters (Zellik, Belgium). Chromatographic separation of the analytes was achieved on an Acquity UPLC^®^ HSS T3 column (100 mm × 2.1 mm I.D., particle size (dp): 1.8 µm) in combination with an Acquity HSS T3 Vanguard pre-column (5 mm × 2.1 mm I.D., dp: 1.8 µm) both from Waters.

Liquid chromatography parameters (mobile phase (MP) composition and gradient programs) were optimized for optimal chromatographic separation of all analytes. The MP for analysis of DON, ^13^C_15_-DON, DOM-1, NIV, ENN B, ^15^N_3_-ENN B, MPA, ^13^C_17_-MPA, ROQ-C, and ^13^C_22_-ROQ-C consisted of 0.01% AA in H_2_O (A) and 0.01% AA in MeOH (B). The MP for the analysis of ZEN, ^13^C_18_-ZEN and the metabolites ZAN, α-ZEL, β-ZEL, α-ZAL, and β-ZAL consisted of H_2_O (A) and ACN (B). A gradient elution was performed as shown in Table 2. The flow-rate was set at 0.3 mL/min. The temperatures of the column oven and autosampler tray were set at 40 °C and 8 °C, respectively. The injection volume was 5 µL/sample.

The UPLC system was coupled to a Xevo^®^ TQ-S MS/MS system, equipped with an ESI probe operating in both the positive and negative ionization mode (all from Waters, Zellik, Belgium). A divert valve was used and the UPLC effluent was directed to the mass spectrometer from 2.50 to 8.50 min for the ESI+ analysis and from 2.20 to 5.20 min for the ESI− analysis. 

Mass spectrometric parameters were optimized by syringe infusion of working solutions of 1 µg/mL of each analyte (flow-rate: 10 µL/min) in combination with the mobile phase (50% A, 50% B, flow-rate: 0.3 mL/min). The following parameters were used: capillary voltage: 3.3 kV (ESI+) and 2.7 kV (ESI−), source offset: 40 V (ESI+) and 50 V (ESI−), desolvation temperature: 400 °C, desolvation gas: 600 L/h, cone gas: 150 L/h, source temperature: 150 °C, nebulizer pressure: 7.0 bar, LM resolution 1 and 2: 3.0, HM resolution 1 and 2: 15.0, ion energy 1: 0.8 (ESI+) and 0.5 (ESI−), ion energy 2: 1.0 (ESI+) and 0.5 (ESI−), collision gas flow: 0.20 mL/min (ESI+) and 0.25 mL/min (ESI−).

MS/MS acquisition was performed in the multiple reaction monitoring (MRM) mode for both the ESI+ and ESI− mode. In Table 1, an overview is given of compound specific parameters such as MRM transitions, cone voltage (CV), collision energy (CE), and retention time (RT). Dwell times were set at 10–50 msec/transition.

MassLynx version 4.1 software was used for data processing (Copyright^©^ 2012, Waters, Zellik, Belgium).

### 4.7. In-House Method Validation

The developed UPLC-MS/MS method was validated in-house by a set of parameters that were in compliance with the recommendations as defined by the European Community [43] and with reference guidelines defined in other EU and FDA documents [36,44,45]. The following parameters have been evaluated for the full method validation with rumen fluid as matrix: linearity, within-run, and between-run accuracy, within-run and between-run precision, limit of quantification (LOQ), limit of detection (LOD), specificity, carry-over, extraction recovery and signal suppression/enhancement. For the cross-validation with rumen fluid + maize silage (10 mg maize silage per mL rumen fluid–buffer mixture) as matrix following parameters have been evaluated: linearity, within-run accuracy, within-run precision, limit of quantification (LOQ), and limit of detection (LOD).

*Linearity*. Calibration curve samples for the full validation and cross-validation were prepared by applying standard working solutions directly onto the homogenized blank samples, followed by a vortex mixing step. Mycotoxins from the calibration curve samples were extracted and quantified as mentioned in Section 4.5 and Section 4.6. By using the same rumen fluid for the calibration curve as the samples to be analyzed, the initial concentration of mycotoxins can be determined by the standard addition method. By adding different concentrations of the mycotoxins to the rumen fluid samples, after measuring the final extract solutions, the observed signals are linearly regressed on the spiked amounts. The original unknown amount is estimated by the opposite of the abscissa intercept of the fitted linear curve. Linearity was evaluated by the correlation coefficients (*r*) and goodness- of-fit coefficients (g) that had to be ≥0.99 and ≤20%, respectively [43]. For a curve where no weighting is applied, the data at the high end of the calibration curve tend to dominate the calculation of the linear regression as the absolute variation is larger for higher concentrations. Hence, 1/x weighted calibration curves were used.

*Accuracy and precision*. Within-run accuracy and precision (repeatability) were determined by analysing six blank samples that were spiked at LOQ, medium, and high concentration levels on the same day. The between-run accuracy and precision were determined by analysing two blank samples that were spiked at LOQ, medium, and high concentration levels, repeated on three different days. The acceptance criteria for accuracy (i.e., the difference between spiked and measured concentration) were −30% to +10% for concentrations between 1 and 10 ng/mL and −20% to +10% for concentrations ≥10 ng/mL. For the precision, the relative standard deviation (RSD) had to be below RSD_max_ = 2^(1−0.5log Conc)^ × 2/3 for within-run precision and 2^(1−0.5log Conc)^ for between-run precision [43,45].

*Limit of quantification*. The limit of quantification (LOQ) was the lowest concentration for which the method was validated with an accuracy and precision that fell within the recommended ranges. The LOQ was determined by analysing six spiked samples and was also the lowest point of the calibration curve.

*Limit of detection*. The limit of detection (LOD) was the lowest concentration that could be determined with a signal-to-noise ratio (S/N) of ≥3 and was calculated using samples spiked at the LOQ level.

*Specificity*. The specificity checks for interferences from endogenous compounds. The S/N ratio of a possible interfering peak in a blank sample (*n* = 1) had to be below the LOD of the analyte in the same elution zone.

*Carry-over*. The carry-over was evaluated by analyzing a solvent sample just after the highest calibrator sample. The eventual analyte concentration in the solvent sample had to be below the LOD.

**Figure 5:**
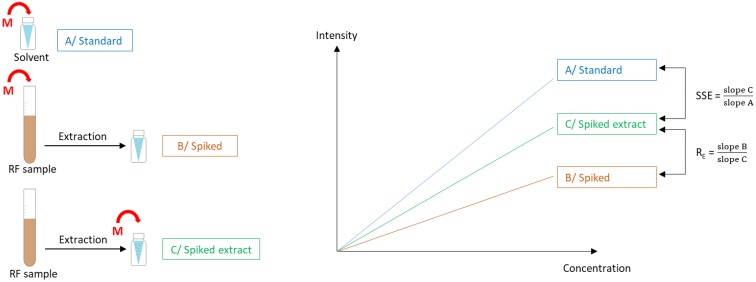
Determination of signal suppression/enhancement (SSE) and extraction recovery (R_E_) in rumen fluid (RF) samples. Three types of calibration curves were prepared by adding mycotoxins (M) to: A/ solvent (standard), B/ rumen fluid before extraction (spiked), C/ rumen fluid samples after extraction (spiked extract). SSE was determined by dividing the slope of the calibration curve for spiked extract by the slope of the calibration curve for standard solutions. Extraction recovery was determined by dividing the slope of the spiked calibration curve by the slope of the calibration curve for spiked extract.

*Extraction recovery (R_E_) and signal suppression*/*enhancement (SSE)*. These effects were determined according to Matuszewski et al. [46] and are visualized in Figure 5. Two types of matrix-matched calibration curves were prepared for each analyte by spiking the blank calibrator samples before (= spiked) and after (= spiked extract) extraction. In addition, one calibration curve was prepared using standard solutions (= standard). The extraction recovery (R_E_) was determined by dividing the slopes of the resulting linear, spiked calibration curves by the related slopes of the calibration curves for spiked extracts. The signal suppression/enhancement (SSE) was evaluated by dividing the slopes of the calibration curves for spiked extracts by the related slopes of the calibration curves for standard solutions.

### 4.8. Evaluation of the Impact of Maize Silage on the Extraction Recovery (R_E_) and Signal Suppression/Enhancement (SSE)

Ten milligram of maize silage was added per mL of rumen fluid–buffer mixture, R_E_ and SSE were determined as mentioned under Section 4.7 and compared with R_E_ and SSE values when using rumen fluid–buffer mixture alone.

## Figures and Tables

**Table 1:** Overview of the multiple reaction monitoring (MRM) transitions and tandem mass spectrometry (MS/MS) parameters for the target analytes.

Analyte	Precursor Ion (*m*/*z*) ^a^	Product Ions (*m*/*z*)	CV ^b^ (V)	CE ^c^ (eV)	RT ^d^ (min)
DON	297.2 [M+H]^+^	248.9 231.0	20	10 12	4.06
^13^C_15_-DON	312.0 [M+H]^+^	263.0 245.2	20	10 10	4.06
DOM-1	281.0 [M+H]^+^	233.0 215.0	27	10 10	4.66
NIV	313.1 [M+H]^+^	175.0	35	15	3.49
ENN B	640.2 [M+H]^+^	196.2 213.8	70	20 22	8.04
^15^N_3_-ENN B	643.3 [M+H]^+^	197.1 215.3	70	23 23	8.04
MPA	321.1 [M+H]^+^	207.0 159.0	25	22 33	6.43
^13^C_17_-MPA	338.0 [M+H]^+^	320.0 218.0	26	10 22	6.43
ROQ-C	390.2 [M+H]^+^	192.9 322.0	32	25 20	5.94
^13^C_22_-ROQ-C	412.0 [M+H]^+^	201.0 339.0	32	27 18	5.94
ZEN	317.3 [M−H]^−^	175.0 131.0	15	25 30	4.95
^13^C_18_-ZEN	335.3 [M−H]^−^	185.1 169.1	15	25 32	4.95
ZAN	319.2 [M−H]^−^	275.2 205.1	20	20 22	4.90
α-ZEL	319.2 [M−H]^−^	275.2 301.0	20	20 22	4.37
β-ZEL	319.2 [M−H]^−^	275.2 301.0	20	20 22	3.99
α-ZAL	321.2 [M−H]^−^	277.1 303.1	30	22 20	4.29
β-ZAL	321.8 [M−H]^−^	277.1 303.1	30	22 20	3.92

^a^*m*/*z* = mass to charge ratio; ^b^ CV = cone voltage; ^c^ CE = collision energy; ^d^ RT = retention time. DON = deoxynivalenol, DOM-1 = deepoxy-deoxynivalenol, NIV = nivalenol, ENN B = enniatin B, MPA = mycophenolic acid, ROQ-C = roquefortine C, ZEN = zearalenone, ZAN = zearalanone, α-ZEL = α-zearalenol, β-ZEL = β-zearalenol, α-ZAL = α-zearalanol and β-ZAL = β-zearalanol. The underlined product ion is used for quantification.

**Table 2:** Overview of the UPLC gradient programs used for the mycotoxin ultra-performance liquid chromatography tandem mass spectrometry (UPLC-MS/MS) methods in ESI+ and ESI− mode.

**Analyte(s)** **ESI+**	**Time** **(min)**	**MP A:B Ratio (*v*:*v*)**
DON, ^13^C_15_-DON, DOM-1, NIV, ENN B, ^15^N_3_-ENN B, MPA, ^13^C_17_-MPA, ROQ-C, ^13^C_22_-ROQ-C	0.00–0.50 0.50–5.50 5.50–7.50 7.50–7.70 7.70–10.0	95:5 Linear to 5:95 5:95 Linear to 95:5 95:5
**Analyte(s)** **ESI−**	**Time** **(min)**	**MP C:D Ratio (*v*:*v*)**
ZEN, ^13^C_18_-ZEN, ZAN, α-ZEL, β-ZEL, α-ZAL, β-ZAL	0.00–0.50 0.50–3.50 3.50–4.90 4.90–5.00 5.00–7.00	70:30 Linear to 30:70 30:70 Linear to 70:30 70:30

Note: ESI+: positive electrospray ionization mode; ESI-: negative ESI; MP: mobile phase. MP A = 0.01% acetic acid (*v*/*v*) in H_2_O; MP B = 0.01% acetic acid (*v*/*v*) in MeOH; MP C = H_2_O; MP D = ACN. DON = deoxynivalenol, DOM-1 = deepoxy-deoxynivalenol, NIV = nivalenol, ENN B = enniatin B, MPA = mycophenolic acid, ROQ-C = roquefortine C, ZEN = zearalenone, ZAN = zearalanone, α-ZEL = α-zearalenol, β-ZEL = β-zearalenol, α-ZAL = α-zearalanol and β-ZAL = β-zearalanol.

**Table 3:** Mycotoxin concentrations (limit of quantification (LOQ), medium and high concentration level) and concentration range in the rumen fluid of the calibration curve (*n* = 10) used for the in-house validation of the UPLC MS/MS method and validation results for linearity (*r* and GOF) and sensitivity (LOD (limit of detection) and LOQ; *n* = 6). For all components, the r and GOF met the acceptance criteria.

Analyte	Calibration Curve Range (ng/mL)	LOD (ng/mL)	LOQ (ng/mL)	Medium (ng/mL)	High (ng/mL)	*r*	GOF (%)
DON	0.45–180	0.05	0.45	12	120	0.9996	4.83
DOM-1	1.56–180	0.08	1.56	12	120	0.9995	4.67
NIV	36–600	5.43	36	120	600	0.9991	4.44
ENN B	0.39–15	<0.01	0.39	1.4	10	0.9982	6.36
MPA	0.6–90	0.17	0.60	6.0	60	0.9995	8.93
ROQ-C	0.1–30	<0.01	0.10	2.0	20	0.9996	5.29
ZEN	0.3–45	0.02	0.30	3.0	30	0.9993	7.82
ZAN	0.3–45	0.07	0.30	3.0	30	0.9997	5.02
α-ZEL	0.3–45	0.08	0.30	3.0	30	0.9990	6.24
β-ZEL	0.3–45	0.07	0.30	3.0	30	0.9938	7.78
α-ZAL	0.3–45	0.02	0.30	3.0	30	0.9983	4.82
β-ZAL	0.3–45	0.02	0.30	3.0	30	0.9931	11.15

Note: DON = deoxynivalenol, DOM-1 = deepoxy-deoxynivalenol, NIV = nivalenol, ENN B = enniatin B, MPA = mycophenolic acid, ROQ-C = roquefortine C, ZEN = zearalenone, ZAN = zearalanone, α-ZEL = α-zearalenol, β-ZEL = β-zearalenol, α-ZAL = α-zearalanol, and β-ZAL = β-zearalanol. Acceptability ranges: *r* = correlation coefficient ≥0.99; GOF = goodness-of-fit coefficient ≤20%. LOD: signal-to-noise ratio (S/N) = 3; LOQ value, lowest point of calibration curve and accuracy and precision within acceptability ranges [36].

**Table 4:** Validation results for within-run precision (*n* = 6) and between-run precision (*n* = 2 × 3) with corresponding accuracy at low (LOQ), medium, and high concentration level. The different concentration levels for each mycotoxin are mentioned in Table 3. For all components, the acceptability ranges for accuracy and precision were met.

Analyte	Within-Run (*n* = 6)						Between-Run (*n* = 2 × 3)					
	Accuracy (%)			Precision (RSD, %)			Accuracy (%)			Precision (RSD, %)		
	LOQ	Medium	High	LOQ	Medium	High	LOQ	Medium	High	LOQ	Medium	High
DON	19.4 ^A^	−0.8 ^C^	1.0 ^C^	5.2 ^A^	2.8 ^C^	1.6 ^D^	19.5 ^A^	−1.6 ^C^	0.4 ^C^	5.2	2.5	1.8
DOM-1	3.7 ^B^	−1.8 ^C^	−5.9 ^C^	5.5 ^B^	5.2 ^C^	9.2 ^D^	2.6 ^B^	−4.7 ^C^	−2.9 ^C^	5.2	5.8	6.6
NIV	4.2 ^C^	−7.6 ^C^	−7.2 ^C^	5.0 ^C^	7.6 ^D^	7.7 ^D^	1.8 ^C^	−8.7 ^C^	−6.1 ^C^	6.8	7.8	6.5
ENN B	−14.0 ^A^	3.6 ^B^	−5.8 ^C^	5.0 ^A^	2.0 ^B^	2.2 ^C^	−14.0 ^A^	1.1 ^B^	−3.8 ^C^	5.0	2.4	3.9
MPA	3.2 ^A^	−8.2 ^B^	5.2 ^C^	5.3 ^A^	2.9 ^B^	4.5 ^C^	4.5 ^A^	−5.1 ^B^	0.5 ^C^	15.5	4.7	5.8
ROQ-C	4.7 ^A^	−2.5 ^B^	−4.9 ^C^	4.5 ^A^	2.0 ^B^	1.3 ^C^	6.8 ^A^	−3.1 ^B^	−4.5 ^C^	4.2	2.4	2.5
ZEN	−5.6 ^A^	2.0 ^B^	−3.9 ^C^	16.6 ^A^	6.2 ^B^	1.5 ^C^	−0.3 ^A^	−3.1 ^B^	0.5 ^C^	10.3	4.5	2.4
ZAN	3.6 ^A^	−2.4 ^B^	−6.5 ^C^	11.1 ^A^	5.2 ^B^	3.9 ^C^	-0.6 ^A^	−0.5 ^B^	−2.0 ^C^	12.4	4.4	5.3
α-ZEL	−7.6 ^A^	−2.4 ^B^	−10.6 ^C^	9.4 ^A^	5.9 ^B^	1.1 ^C^	−9.2 ^A^	−0.6 ^B^	−5.1 ^C^	12.4	5.9	8.2
β-ZEL	−4.6 ^A^	−19.3 ^B^	4.6 ^C^	8.6 ^A^	12.0 ^B^	6.3 ^C^	−2.8 ^A^	−8.6 ^B^	−5.8 ^C^	8.2	13.4	14.5
α-ZAL	−21.3 ^A^	−5.0 ^B^	1.8 ^C^	14.5 ^A^	9.2 ^B^	2.9 ^C^	−13.5 ^A^	−1.9 ^B^	−5.5 ^C^	16.9	10.1	9.6
β-ZAL	−6.7 ^A^	−10.2 ^B^	−9.9 ^C^	16.4 ^A^	12.0 ^B^	10.9 ^C^	−6.9 ^A^	−5.3 ^B^	−7.1 ^C^	16.8	14.3	14.9

Note: DON = deoxynivalenol, DOM-1 = deepoxy-deoxynivalenol, NIV = nivalenol, ENN B = enniatin B, MPA = mycophenolic acid, ROQ-C = roquefortine C, ZEN = zearalenone, ZAN = zearalanone, α-ZEL = α-zearalenol, β-ZEL = β-zearalenol, α-ZAL = α-zearalanol, and β-ZAL = β-zearalanol. Acceptability ranges for accuracy: −50 to +20% for conc. <1 ng/mL (A in superscript), −30 to +10% for conc. ≥1 to 10 ng/mL (B in superscript), −20% to +10% for conc. ≥10 ng/mL (C in superscript), within-run precision: RSD_max_ = 30% for conc. <1 ng/mL (A in superscript), RSD_max_ = 25% for conc. ≥1 to 10 ng/mL (B in superscript), RSD_max_ = 15% for conc. ≥10 ng/mL to 100 ng/mL (C in superscript), RSD_max_ = 10% for conc. ≥100 ng/mL (D in superscript), between-run precision: RSD_max_ = 2^(1-0.5logConc).^

**Table 5:** Extraction recovery (R_E_) and signal suppression/enhancement (SSE) of mycotoxins in the rumen fluid–buffer mixture (Matrix A: RF) and in the rumen fluid–buffer mixture with 10 mg maize silage per mL (Matrix B: RF + MS). The results show that SSE and R_E_ differ between different matrices. Hence, matrix-matched calibration curves are needed when analyzing samples.

Analyte	Matrix A: RF		Matrix B: RF + MS	
	SSE (%)	R_E_ (%)	SSE (%)	R_E_ (%)
DON	79	42	55	44
NIV	68	9	60	8
ENN B	241	13	197	16
MPA	45	2	48	3
ROQ-C	69	16	62	16
ZEN	60	19	52	19
DOM-1	71	46	60	48
α-ZEL	60	20	53	21
β-ZEL	67	21	60	25
ZAN	64	19	53	21
α-ZAL	64	22	58	25
β-ZAL	68	23	63	29

Note: DON = deoxynivalenol, DOM-1 = deepoxy-deoxynivalenol, NIV = nivalenol, ENN B = enniatin B, MPA = mycophenolic acid, ROQ-C = roquefortine C, ZEN = zearalenone, ZAN = zearalanone, α-ZEL = α-zearalenol, β-ZEL = β-zearalenol, α-ZAL = α-zearalanol, and β-ZAL = β-zearalanol. SSE = slope_(spiked extract calibration curve)_/slope_(standard calibration curve)_, R_E_ = slope_(spiked calibration curve)_/slope_(spiked extract calibration curve)_.

**Table 6:** Mycotoxin concentrations (limit of quantification (LOQ), medium and high concentration level) and concentration range of the calibration curve (*n* = 10) used for the in-house cross-validation of the UPLC MS/MS method with rumen fluid–buffer mixture and maize silage (10 mg/mL) and validation results for linearity (r and GOF) and sensitivity (LOD (limit of detection) and LOQ; *n* = 6). For all components, the r and GOF met the acceptance criteria.

Analyte	Calibration Curve Range (ng/mL)	LOD (ng/mL)	LOQ (ng/mL)	Medium (ng/mL)	High (ng/mL)	*r*	GOF (%)
DON	8–187	0.41	7.99	19.4	120	0.9998	1.65
DOM-1	6–180	0.35	6.00	12.0	120	0.9993	3.22
NIV	36–900	9.28	36	120	600	0.9974	6.64
ENN B	0.44–15.34	0.01	0.44	1.34	10	0.9985	5.81
MPA	1.20–90	0.42	1.20	6.0	60	0.9986	6.55
ROQ-C	0.2–30	0.01	0.20	2.0	20	0.9978	9.29
ZEN	1.06–45	0.04	1.06	3.5	30	0.9995	4.09
ZAN	0.3–45	0.10	0.30	3.0	30	0.9989	5.58
α-ZEL	0.6–45	0.15	0.60	3.0	30	0.9994	5.24
β-ZEL	0.6–45	0.08	0.60	3.0	30	0.9992	6.57
α-ZAL	1.5–45	0.04	1.50	3.0	30	0.9993	4.91
β-ZAL	0.3–45	0.06	0.30	3.0	30	0.9994	5.83

Note: DON = deoxynivalenol, DOM-1 = deepoxy-deoxynivalenol, NIV = nivalenol, ENN B = enniatin B, MPA = mycophenolic acid, ROQ-C = roquefortine C, ZEN = zearalenone, ZAN = zearalanone, α-ZEL = α-zearalenol, β-ZEL = β-zearalenol, α-ZAL = α-zearalanol, and β-ZAL = β-zearalanol. Acceptability ranges: *r* = correlation coefficient ≥0.99; GOF = goodness-of-fit coefficient ≤20%. LOD: signal-to-noise ratio (S/N) = 3; LOQ value, lowest point of calibration curve and accuracy and precision within acceptability ranges [36].

**Table 7:** Validation results of the in-house cross-validation of the UPLC MS/MS method with rumen fluid–buffer mixture and maize silage (10 mg/mL) for within-run precision (*n* = 6) with corresponding accuracy at low (LOQ), medium, and high concentration level. The different concentration levels for each mycotoxin are mentioned in Table 6. For all components, the acceptability ranges for accuracy and precision were met.

Analyte	Within-Run (*n* = 6)					
	Accuracy (%)			Precision (RSD, %)		
	LOQ	Medium	High	LOQ	Medium	High
DON	−0.3 ^B^	0.1 ^C^	−4.8 ^C^	0.6 ^B^	2.5 ^C^	3.6 ^D^
DOM-1	−7.9 ^B^	−6.3 ^C^	1.9 ^C^	9.7 ^B^	3.3 ^C^	4.7 ^D^
NIV	1.1 ^C^	5.5 ^C^	−1.0 ^C^	5.6 ^C^	2.8 ^D^	8.0 ^D^
ENN B	−2.0 ^A^	4.5 ^B^	−8.5 ^C^	4.0 ^A^	2.9 ^B^	5.1 ^C^
MPA	−1.1 ^B^	−7.3 ^B^	3.6 ^C^	5.0 ^B^	8.9 ^B^	7.4 ^C^
ROQ-C	−12.5 ^A^	−1.0 ^B^	−5.2 ^C^	3.1 ^A^	3.3 ^B^	3.0 ^C^
ZEN	−6.0 ^B^	2.5 ^B^	3.1 ^C^	8.8 ^B^	6.2 ^B^	7.3 ^C^
ZAN	−2.2 ^A^	0.9 ^B^	8.3 ^C^	6.0 ^A^	4.7 ^B^	0.9 ^C^
α-ZEL	1.9 ^A^	−7.6 ^B^	−5.2 ^C^	4.7 ^A^	2.9 ^B^	6.5 ^C^
β-ZEL	−1.4 ^A^	−17.6 ^B^	−0.7 ^C^	8.2 ^A^	10.0 ^B^	7.8 ^C^
α-ZAL	−10.9 ^B^	−17.0 ^B^	7.4 ^C^	7.8 ^B^	9.5 ^B^	6.7 ^C^
β-ZAL	−8.3 ^A^	−16.8 ^B^	−2.9 ^C^	6.4 ^A^	11.1 ^B^	7.5 ^C^

Note: DON = deoxynivalenol, DOM-1 = deepoxy-deoxynivalenol, NIV = nivalenol, ENN B = enniatin B, MPA = mycophenolic acid, ROQ-C = roquefortine C, ZEN = zearalenone, ZAN = zearalanone, α-ZEL = α-zearalenol, β-ZEL = β-zearalenol, α-ZAL = α-zearalanol, and β-ZAL = β-zearalanol. Acceptability ranges for accuracy: −50 to +20% for conc. <1 ng/mL (A in superscript), −30 to +10% for conc. ≥1 to 10 ng/mL (B in superscript), −20% to +10% for conc. ≥10 ng/mL (C in superscript), and within-run precision: RSD_max_ = 30% for conc. <1 ng/mL (A in superscript), RSD_max_ = 25% for conc. ≥1 to 10 ng/mL (B in superscript), RSD_max_ = 15% for conc. ≥10 ng/mL to 100 ng/mL (C in superscript), RSD_max_ = 10% for conc. ≥100 ng/mL (D in superscript).

## Supplementary Materials

The following are available online at https://www.mdpi.com/2072-6651/11/9/519/s1, Table S1: Mycotoxins found in maize samples in Belgium, Table S2: Mycotoxins found in silage samples in Europe.
