# Supplementary material for: Development of an UPLC-MS/MS Method for the Analysis of Mycotoxins in Rumen Fluid with and without Maize Silage Emphasizes the Importance of Using Matrix-Matched Calibration
Source: Toxins (Basel). 2019 Sep 7;11(9):519. doi: 10.3390/toxins11090519 (PMC6784025; doi:10.3390/toxins11090519)
Supplement: Supplementary file 1 [file toxins-11-00519-s001.pdf]

Supplementary Materials: Development of an UPLC-MS/MS Method for the Analysis of Mycotoxins in Rumen Fluid with and without Maize Silage Emphasizes the Importance of Using Matrix-Matched Calibration

Sandra Debevere, Siegrid De Baere, Geert Haesaert, Michael Rychlik, Veerle Fievez and Siska Croubels

Table S1. Mycotoxins found in maize samples in Belgium (Vandicke J. (Department of Plants and Crops, Faculty of Bioscience Engineering, Ghent, Belgium). Personal communication, 2017).

**Mycotoxins in Maize**		**2016 (91 Samples)**	
		**Prevalence (% of all samples)**	**% samples exceeding EU Regulation ***
Nivalenol	NIV	98.9	/
Deoxynivalenol	DON	92.3	2
Zearalenone	ZEN	64.8	1
EnniatinB	ENN B	42.9	/
Diacetoxyscirpenol	DAS	11.0	/
Fumonisin B1	FB1	2.5	0
Alternariolmethylether	AME	2.2	/
T-2 toxin	T-2	1.1	0
Sterigmatocystin	STERIG	1.1	/
Alternariol	AOH	0	/
Fumonisin B2	FB2	0	0
Fumonisin B3	FB3	0	0
Fusarenon-X	FX	0	/
Roquefortin C	ROQ-C	0	/

0% incidence: neosolaniol (NEO), aflatoxin B2 (AFB2), AFB1, HT-2 toxin (HT2), ochratoxin A (OTA) * European Commission Commission Recommendation No 2006/576/EC of 17 August 2006 on the presence of deoxynivalenol, zearalenone, ochratoxin A, T-2 and HT-2 and fumonisins in products intended for animal feeding. Off. J. Eur. Union 2006, L229, 7–9. When no number is mentioned (/), no EU regulation exists for this mycotoxin.

Table S2. Mycotoxins found in silage samples in Europe.

Mycotoxins in Maize Silage (21 Samples)		Prevalence (% of 21 Maize Silage Samples in Belgium) [1–3]		Prevalence (% of 100 Silage Samples in Europe) [4]
		Non-Moldy Parts	Moldy Hot Spots	
Deoxynivalenol	DON	100	100	100
Roquefortin C	ROQ-C	62	100	36
Citrinin	CIT	95	95	
Mycophenolic acid	MPA	95	86	
Zearalenone	ZEN	90	90	32
Enniatin B	ENN B	86	86	91
Nivalenol	NIV			64
HT-2 toxin	HT-2	81	86	3
Ochratoxin A	OTA	62	72	4
Mevalonic acid	MVA	29	52	
Patulin	PAT	29	38	
Gliotoxin	GT	14	0	
Penitrem A	PA	0	19	

References

1. Tangni, E. K.; Pussemier, L.; Bastiaanse, H.; Haesaert, G.; Foucart, G.; Van Hove, F. Presence of mycophenolic acid , roquefortine C , citrinin and ochratoxin A in maize and grass silages supplied to dairy cattle in Belgium. *J. Anim. Sci. Adv.* **2013**, 3, 598–612.
2. Driehuis, F.; Spanjer, M. C.; Scholten, J. M.; te Giffel, M. C. Occurrence of mycotoxins in feedstuffs of dairy cows and estimation of total dietary intakes. *J. Dairy Sci.* **2008**, 91, 4261–4271.
3. Wambacq, E.; Vanhoutte, I.; Audenaert, K.; Gelder, L. De; Haesaert, G. Occurrence, prevention and remediation of toxigenic fungi and mycotoxins in silage: a review. *J. Sci. Food Agric.* **2016**, *96*, 2284–2302.
4. De Boevre, M. (Centre of Excellence in Mycotoxicology and Public Health, Department of Bioanalysis, Ghent, Belgium). Personal communication, 2016.
